# Periostin-expressing cell-specific transforming growth factor-β inhibition in pulmonary artery prevents pulmonary arterial hypertension

**DOI:** 10.1371/journal.pone.0220795

**Published:** 2019-08-22

**Authors:** Mitsuru Seki, Nozomi Furukawa, Norimichi Koitabashi, Masaru Obokata, Simon J. Conway, Hirokazu Arakawa, Masahiko Kurabayashi

**Affiliations:** 1 Department of Pediatrics, Gunma University Graduate School of Medicine, Maebashi, Gunma, Japan; 2 Department of Cardiovascular Medicine, Gunma University Graduate School of Medicine, Maebashi, Gunma, Japan; 3 Department of Pediatrics, Jichi Medical University, Shimotsuke, Tochigi, Japan; 4 Herman B. Wells Center for Pediatric Research, Indiana University School of Medicine, Indianapolis, Indiana, United States of America; Ann and Robert H Lurie Children's Hospital of Chicago, Northwestern University, UNITED STATES

## Abstract

Transforming growth factor beta (TGF-β) has been shown to play a critical role in pathogenesis of pulmonary arterial hypertension (PAH) although the precise role of TGF-β signaling remains uncertain. A recent report has shown that periostin (Pn) is one of the most upregulated proteins in human PAH lung compared with healthy lungs. We established type I TGF-β receptor knockout mice specifically with Pn expressing cell (Pn-Cre/*Tgfb1*^fl/fl^ mice). Increases in PA pressure and pulmonary artery muscularization were induced by hypoxia of 10% oxygen for 4 weeks. Lung Pn expression was markedly induced by 4 week-hypoxia. Pn-Cre/*Tgfb1*^fl/fl^ mice showed lower right ventricular pressure elevation, inhibition of PA medial thickening. Fluorescent co-immunostaining showed that Smad3 activation in Pn expressing cell is attenuated. These results suggest that TGF-β signaling in Pn expressing cell may have an important role in the pathogenesis of PAH by controlling medial thickening.

## Introduction

Pulmonary arterial hypertension (PAH) is a rare disease of the small pulmonary arteries (PAs) characterized by raised pulmonary artery resistance, which results in a marked increase in pulmonary arterial pressure, remodeling and right ventricular hypertrophy[1]. The increase in pulmonary vascular resistance is due to adventitial, medial, and intimal thickening of PAs that results from proliferation and migration of smooth muscle cell, proliferation or activation of fibroblasts, and proliferation of disorganized endothelial cell[2]. Since the consequence of increased right ventricle afterload might be critical, it is necessary to clarify the pathophysiology of PAH to develop further therapies.

Periostin (Pn), also termed osteoblast-specific factor 2, is a matricellular proteins that was initially suggested to function as a cell adhesion molecule for preosteoblasts[3]. Subsequent studies have shown that Pn can act as a ligand affecting cell migration, adhesion, and epithelial-mesenchymal transition in various state[4,5,6]. Pn is also involved in tissue remodeling and wound healing, and in interacting with other extracellular matrix proteins (ECM) such as the transition of fibroblasts to myofibroblasts, collagen fibrillogenesis, and ECM synthesis[7,8]. Recently, in a rat hypoxia-induced PAH model, Pn expression was found to increase in both lung and isolated pulmonary artery smooth muscle cells (PASMCs) in response to hypoxic stress[9]. In addition, proteomic analysis of lung tissues from patients with PAH showed that Pn was one of the most upregulated proteins in lung with PAH compared with control samples, and is localized in the neointima of “remodeled” PA, while normal PA had no Pn expression[10]. Therefore, Pn might be involved in the pathogenesis of PAH.

Transforming growth factor (TGF)-β signaling is critical in the pathogenesis of PAH, and its inhibition prevents the PAH progression[11]. Gene abnormalities in bone morphogenetic protein type II receptor, a member of TGF-β cell-signaling superfamily, have been reported to be involved in the development of PAH[12,13]. These preclinical studies indicate that targeting TGF-β signaling may be promising therapy for PAH, however, systemic inactivation of TGF-β signaling has raised a number of safety concerns. Systemic knockout mice of type I TGF-β receptor (*Tgfbr1*) has been shown to be embryonic lethal[14]. Homozygous deletion of *Tgf-β1* in mice leads to death in utero or within the first 3–4 weeks after birth due to multiorgan inflammation[15]. Therefore, cell-specific or organ-specific investigation is important to optimize TGF-β-targeting therapeutic strategy[16].

To elucidate the pathogenesis of PAH with focusing TGF-β signaling, we used the Cre-loxP system. Recently, Pn promoter-Cre transgenic mice (Pn-Cre) was established by Conway and coworkers and used for fibroblast-specific gene deletion studies[17,18]. Pn-Cre mice might be available for conditional gene-knockdown in remodeled PA under hypoxia condition; that is, Pn expression might increase only in remodeled PA of hypoxia-induced PAH mice. Since Pn is an inducible protein in remodeled PA, we examine the role of Pn-expressing cells and TGF-β signaling in the pathogenesis of PAH.

## Materials and methods

### Animal experiments

All animal experiments were performed in accordance with the protocols approved by the Committee of Experimental Animal Research of Gunma University (Permit Number; 11–027). Hypoxia-induced PH models were used to assess the development of PH in mice. Eight-week-old male wild-type (WT) mice on a normal chow diet were exposed to hypoxia (10% O_2_) or normoxia for 4 weeks as previously described[19,20]. Briefly, hypoxic mice were housed in an acrylic chamber with a nonrecirculating gas mixture of 10% O_2_ and 90% N_2_ by adsorption-type oxygen concentrator to utilized exhaust air (Teijin, Tokyo, Japan), whereas normoxic mice were housed in room air (21% O_2_) under a 12-hour light/dark cycle. After 4 weeks of exposure to hypoxia (10% O_2_) or normoxia, mice were anesthetized with isoflurane (1.0%). To examine the development of PH, we measured right ventricular systolic pressure (RVSP), right ventricular hypertrophy (RVH), and pulmonary vascular remodeling. For right heart catheterization, a 1.4-F pressure catheter (SPR-671, Millar Instruments Inc,USA) was inserted in the right jugular vein and advanced into the RV to measure RVSP as described previously[21]. All data were analyzed using the PowerLab data acquisition system (AD Instruments, Australia) and averaged >10 sequential beats.

### Generation of Pn-Cre/*Tgfbr1* ^fl/fl^ mouse and Pn-Cre/lacZ receptor mouse

**Fig 1:**
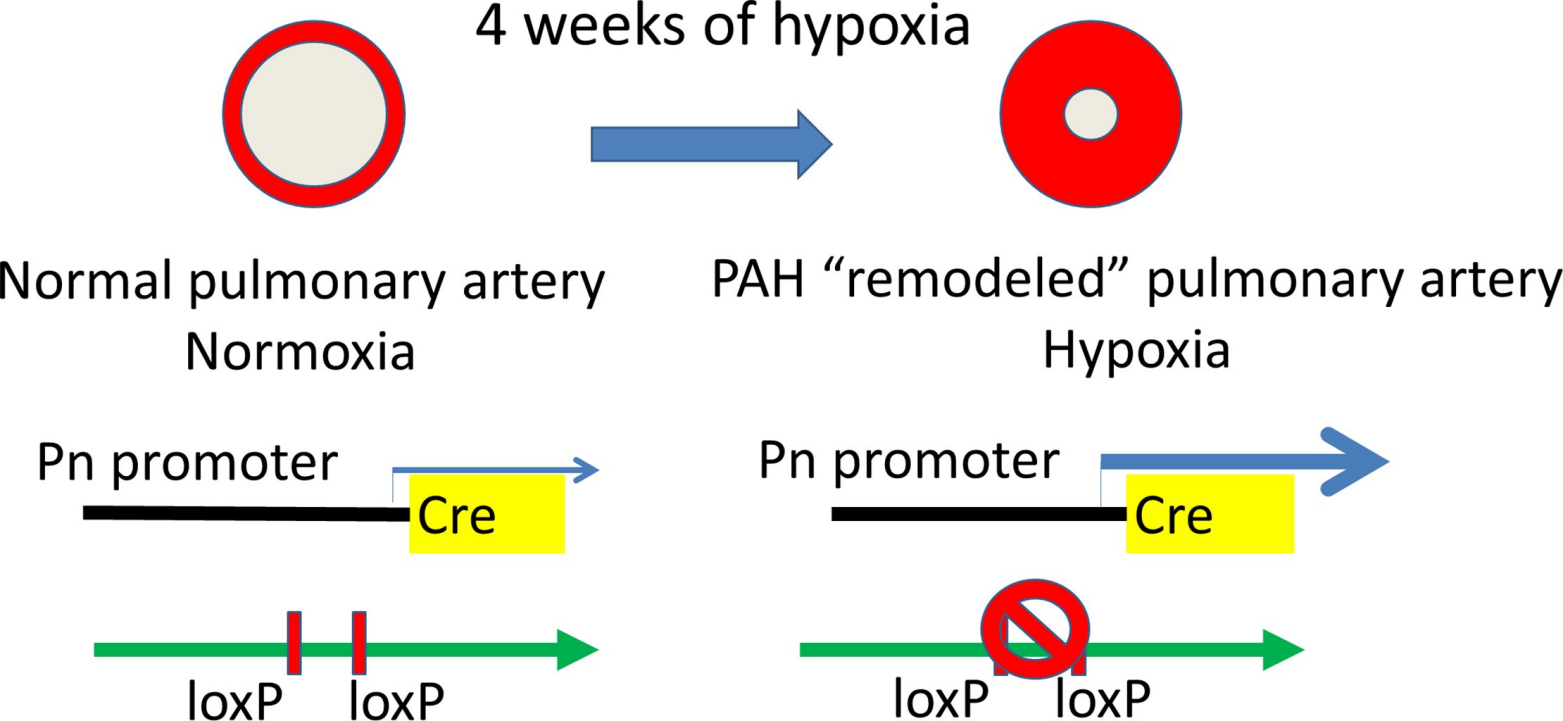
Schema of our hypoxia-induced PAH model in Pn-Cre mice. In this model, we assume that hypoxia-induced Pn-mediated *Cre-loxP* activation will reduce *Tgfbr1* expression in remodeled pulmonary artery and attenuate TGF-β signaling, resulting that Pn-Cre mice being available for conditional gene-knockdown under hypoxia.

C57BL/6 mice (CLEA Japan) were used for control study to examine Pn expression in PAH model. Pn-Cre transgenic mice were obtained from Dr. Conway (Indiana University, USA) [22]. Type I TGF-βreceptor-floxed mice (*Tgfbr1*^fl/fl^) were obtained from Dr. Pinto (University of Amsterdam, Netherlands) [16]. To knockdown of TGF-β signaling in Pn-expressing cells in mouse, Pn-Cre transgenic mice were crossed with *Tgfbr1*^fl/fl^ mice (Pn-Cre/*Tgfbr*1^fl/fl^). Genotype was confirmed with primers to Cre and *Tgfbr1* floxed alleles. All mice were genotyped by polymerase chain reaction amplification of tail-clip samples, and all experiments were performed with male mice using littermate Cre-negative/*Tgfbr1*^fl/fl^ as controls. In this model, we assume that hypoxia-induced Pn-mediated *Cre-loxP* activation will reduce *Tgfbr1* expression in remodeled pulmonary artery and attenuate TGF-β signaling (Fig 1).

To identify Pn-Cre expressing cells, we created Pn-Cre/lacZ reporter mice. Pn-Cre transgenic mice were crossed with ROSA26-lacZ mice. This mouse is LacZ positive for Pn-Cre expressing cells.

### Histological analysis

After hemodynamic measurements, following deep anesthesia with isoflurane, mice were sacrificed by cervical dislocation and the left lungs were ligated and excised immediately. Tissue was frozen rapidly and stored at -70°C until RNA and protein analysis. The heart and right lung were washed with cold phosphate–buffered saline (PBS) and fixed in 10% formaldehyde. The hearts were dissected, and the right ventricle (RV) wall was separated from the left ventricle (LV) and septum. The ratio of the RV to the LV plus septum weight was calculated to determine the extent of RVH[19]. Paraffin sections were stained with Elastica Van Gieson (EVG) staining or used for immunostaining. Pulmonary arteries adjacent to an airway distal to the respiratory bronchiole were evaluated as previously reported[19,20]. Briefly, arteries were considered fully muscularized if they had a distinct double-elastic lamina visible throughout the diameter of the vessel cross section. The percentage of vessels with double-elastic lamina was calculated as the number of muscularized vessels per total number of vessels counted by a computer-assisted imaging system (WinROOF 2013, Mitani Corporation, Tokyo, Japan).

Fluorescence immunohistochemistry was performed as follows; the paraffin embedded sections were stained with goat anti-phospho Smad 2/3 antibody (Ser 423/425; Santa Cruz) using Alexa Fluor 488-conjugated anti-rabbit or anti-goat TSA kit (Thermo Fisher Scientific), respectively, following the manufacturer’s protocol[16]. Rabbit anti-Pn antibody (ab14041: Abcam) and goat anti-TGFBR1 antibody (ab121024: Abcam) were used for Pn and type I TGF-β receptor immunostaining, respectively. DAPI (Thermo Fisher Scientific), WGA (Alexa Fluor 637; Thermo Fisher Scientific), and mouse anti-α smooth muscle actin (SMA) βantibody (M0851:DAKO) were used for counterstaining of nuclei, plasma membrane/extracellular matrix and vascular smooth muscle cells (SMCs), respectively. Confocal analysis was performed on a Zeiss LSM510 and LSM880 laser scanning confocal microscopes[16]. LacZ antibody (ab9361: Abcam) was used for immunostaining of Pn-Cre expressing cells. NG2 (ab5009: Abcam) was used for the pericyte marker and Vimentin (ab8978:Abcam) was used for the fibroblast marker.

### RNA analysis

Total RNA was prepared from mouse whole lung using TRIzol reagent (Thermo Fisher Scientific), following the manufacturer’s protocol. RNA transcript was analyzed by quantitative real-time PCR using SYBR green[16]. Validated real-time PCR primers were obtained from the Perfect Real Time Support System (Takara Bio Inc.). The primers for Pn recognize the longest isoform, isoform 1, which has been shown to have a pathological role in cardiac remodeling[23]. Real-time RT-PCR was performed, recorded, and analyzed using the MX3000P quantitative system (Stratagene, La Jolla, CA).

### Western blots

Tissue samples and cells were homogenized on ice in RIPA buffer (20 mM Tris-HCl (pH 7.4), 150 mM NaCl, 1% NP-40, 1% sodium deoxycholate and 0.1% SDS) containing complete mini and phosSTOP solution (Roche, Mannheim, Germany). The mixture was centrifuged at 15,000xg. for 30 min and the supernatant was subjected to SDS–PAGE. Protein concentrations were determined by the Bradford method using a colorimetric assay (Bio-Rad, Hercules, CA). Western blot analysis was performed according to standard procedures using the following primary antibodies: anti-phospho Smad 2/3 (Ser 423/425; Santa Cruz), anti-Smad 2/3 (Cell Signaling Technology, #3102), anti-phospho Smad1 (Cell Signaling Technology, #9553), anti-Smad1 (Cell Signaling Technology, #9743), and GAPDH (Cell Signaling Technology). Antigens were revealed by Immobilon Western HRP Substrate (Millipore, Billerica, MA) after incubation with horseradish peroxidase-conjugated secondary antibody. The band’s density was quantified using ImageJ software.

### Statistics

All values are expressed as mean ± SEM. Group comparisons were performed by 1- or 2-way analysis of variance (ANOVA) or by unpaired 2-tailed Student’s t test. Sample sizes and individual statistical results for all analyses are provided in the figures.

## Results

### Hemodynamics and histological change in mice models of hypoxia induced pulmonary hypertension

RV catheterization showed that RV pressure and RV weight were significantly increased after 4 weeks hypoxia in control mice (S1A–S1D Fig). Pathologically, 4 weeks of hypoxia resulted in a significant increase of PA muscularization as evaluated by existence of double layer in Elastica van Gieson staining compare to normoxia control mice (S1E and S1F Fig). These findings suggested that our hypoxia-induced PAH mice model was correctly established. Histologically, there was a strong tendency for RV fibrosis in the hypoxia group compared with normoxia group, but it was not statistically significant.

### Hypoxia-induced Pn was mainly expressed in perivascular area in mice

**Fig 2:**
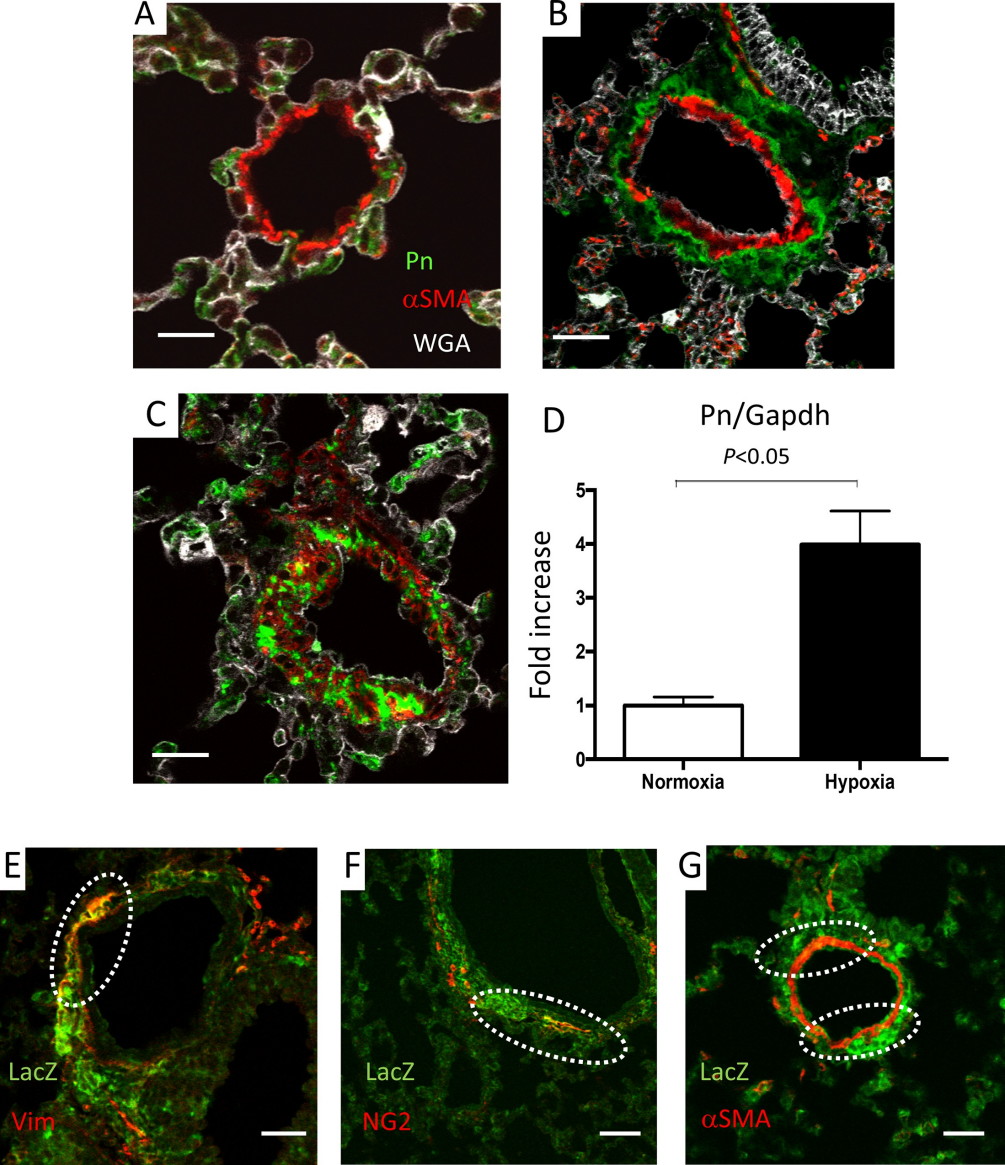
Fluorescent immunostaining with lung tissues of wild-type mice (**A**-**C**). Immunostaining showing that Periostin (Pn) was mainly expressed in airway epithelial cell in normoxia control mice (**A**). Hypoxia-induced Pn was highly expressed in the perivascular area (**B**) and in neointima (**C**). Smooth muscle α actin, αSMA. *Pn* mRNA levels were significantly increased under hypoxia compared to normoxia control mice (a 4.0-fold increase) in lung (n = 4 in each group) (**D**). Group comparisons were performed by unpaired 2-tailed Student’s t test. Immunostaining with lung tissues of Pn-Cre/lacZ reporter mice showing LacZ-positive Pn-Cre-expressing cells (**E**-**G**). Dotted circles show overlap of two proteins. Pn-Cre is partially expressed with vimentin (**E**) and NG2 (**F**). Pn-Cre is partially expressed with α-SMA (**G**). Size bar: 20μm.

Fluorescent immunostaining using wild-type mice showed that Pn is mainly expressed in airway epithelial cell in normoxia control mice (Fig 2A), but hypoxia-induced Pn was highly expressed in perivascular area (Fig 2B) and in neointima (Fig 2C). Its distribution pattern was mainly different with αSMA, but some cells in neointima showed both Pn and αSMA positive (Fig 2C). Pn mRNA level was significantly increased under hypoxia compare to normoxia control mice (a 4.0-fold increase) in mice lung (Fig 2D).

### Characteristics of Pn-Cre-expressing cells

To identify Pn-Cre expressing cells, we created Pn-Cre/lacZ reporter mice. This mouse shows LacZ positive for Pn-Cre expressing cells. Hypoxia-exposure induced lacZ protein (Pn-Cre) expression in lung tissues, especially perivascular area (S2A–S2D Fig. Pn-Cre is partially expressed with vimentin, a fibroblast marker (Fig 2E), NG2, a pericyte marker (Fig 2F), and with αSMA (Fig 2G, S2C and S2D Fig).

### TGF-β Signaling in Pn-Cre/*Tgfbr1*^fl/fl^ mice models under hypoxia

Fig 1 shows the schema of our hypoxia-induced PAH model in Pn-Cre mice. Given that Pn gene expression is induced in pulmonary artery by hypoxia, we assume that hypoxia-induced Pn-mediated *Cre-loxP* activation will reduce *Tgfbr1* expression in remodeled pulmonary artery and attenuate TGF-β signaling (Fig 1).

**Fig 3:**
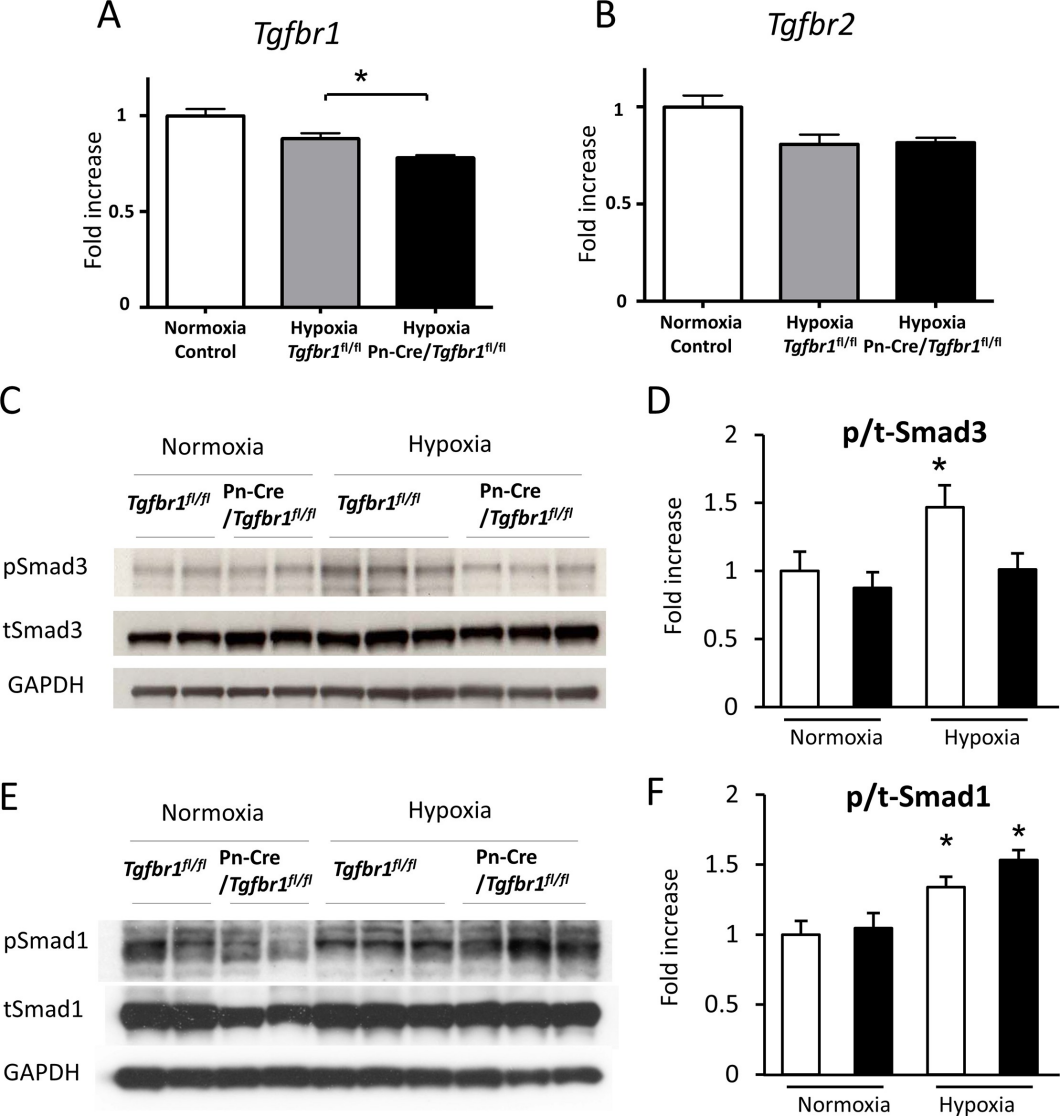
TGF-β signaling inhibition in Pn-Cre/*Tgfbr1*^fl/fl^ mice. Real-time quantitative RT-PCR of lung tissues in control (n = 4), *Tgfbr1*^fl/fl^ (n = 3), and Pn-Cre/*Tgfbr1*^fl/fl^ (n = 3) mice (**A** and **B**). TGF-β receptor type I (**A**), but not type II (**B**) of Pn-Cre/*Tgfbr1*^fl/fl^ was decreased in lung tissue under hypoxic condition. Western blots for Smads of lung tissue in control, *Tgfbr1*^fl/fl^, and Pn-Cre/*Tgfbr1*^fl/fl^ with or without hypoxia exposure (**C-F**). Phosphorylation levels of Smad3 and Smad1 were estimated by the ratio of phospho-Smad ant total Smad band intensities. A representative blot for Smad3 and the summary data (N = 6-8/each) is shown in **C** and **D**, respectively. A representative blot for Smad1 and the summary data (N = 6-8/each) is shown in **E** and **F**, respectively. Group comparisons were performed by 1-way ANOVA.

In fact, TGF-β receptor type I of Pn-Cre/*Tgfbr1*^fl/fl^ was decreased in lung tissue under hypoxic condition according to real-time quantitative RT-PCR (Fig 3A) and TGF-β receptor type II was not decreased in those lung tissues (Fig 3B), suggesting that Pn-Cre mediated recombination is working under hypoxia. Under hypoxia, phosphorylation of Smad3 was increased in *Tgfbr1*^fl/fl^ and suppressed in Pn-Cre/*Tgfbr1*^fl/fl^ (Fig 3C and 3D). On the other hand, phosphorylation of Smad1 was not attenuated in Pn-Cre/*Tgfbr1*^fl/fl^ under hypoxic condition (Fig 3E and 3F).

**Fig 4:**
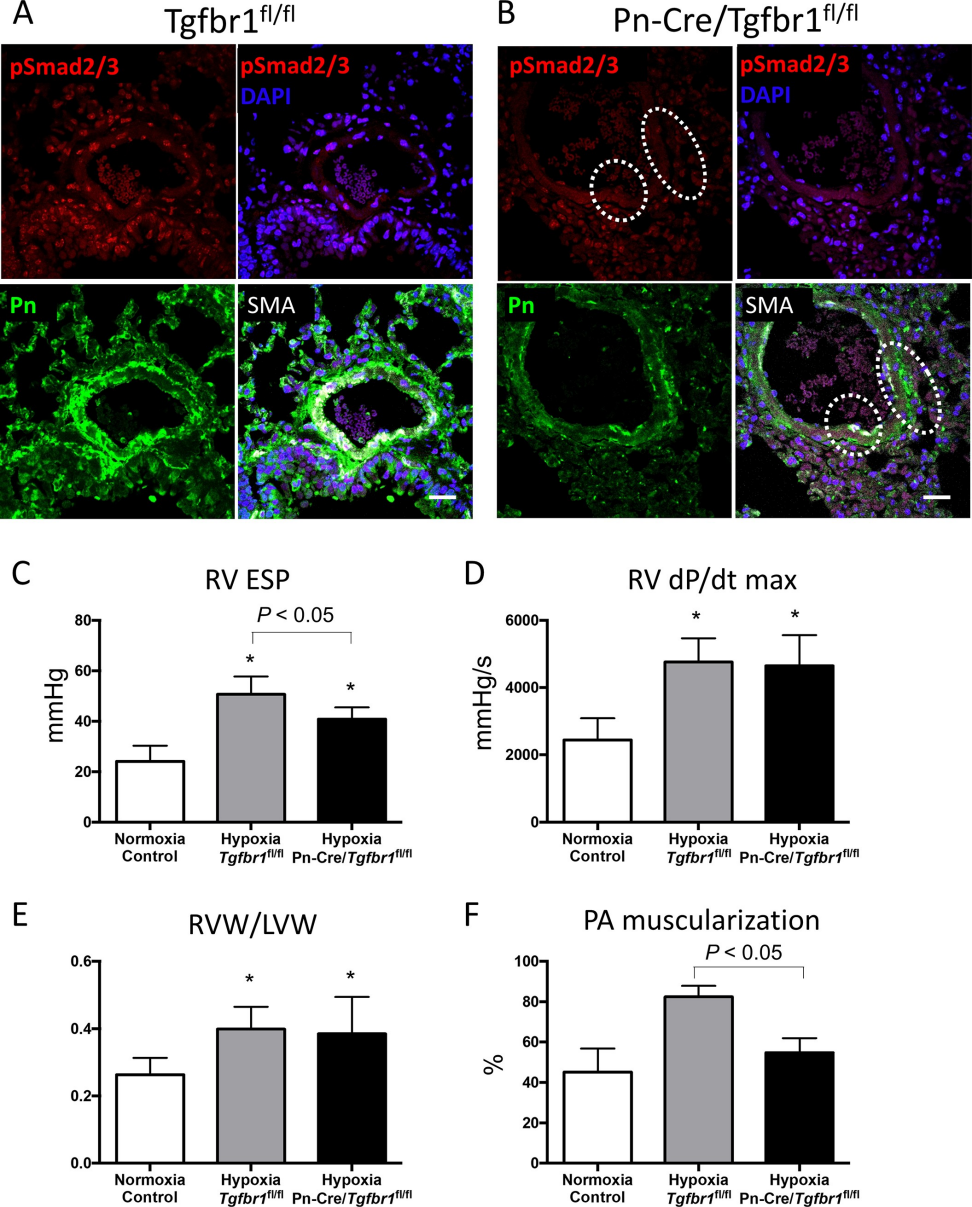
Co-immunostaining with Phosho-Smad2/3, Pn, α-SMA and DAPI in lung tissues of *Tgfbr1*^fl/fl^ mice (**A**) and Pn-Cre/*Tgfbr1*^fl/fl^ mice (**B**) under hypoxia. Immunostaining shows Smad2/3 activation (red) under hypoxic condition in *Tgfbr1*^fl/fl^ mice (**A**). Smad2/3 activation was reduced in Pn-Cre/*Tgfbr1*^fl/fl^ mice especially in Pn-positive area indicated by dotted circles (**B**). Size bar: 20μm. Bar charts show RVESP, RVdP/dtmax, and indices of RV weight (RVW/LVW) in normoxia control (n = 10), hypoxia *Tgfbr1*^fl/fl^ mice (n = 6), and hypoxia Pn-Cre/*Tgfbr1*^fl/fl^ mice (n = 6) (**C**-**E**). RV pressure was significantly decreased in Pn-Cre/*Tgfbr1*^fl/fl^ mice compare to *Tgfbr1*^fl/fl^ mice (**C**). There was no significant difference either in RV dP/dt max or RVW/LVW between the two groups (**D** and **E**). Bar chart **F** shows PA muscularization in normoxia control (n = 9), hypoxia *Tgfbr1*^fl/fl^ mice (n = 4), and hypoxia Pn-Cre/*Tgfbr1*^fl/fl^ mice (n = 4), showing that PA muscularization was significantly attenuated in Pn-Cre/*Tgfbr1*^fl/fl^ mice. Group comparisons were performed by 1-way ANOVA.

Immunostaining showed Smad2/3 was activated in various cells under hypoxic condition (Fig 4A). In *Tgfbr1* ^fl/fl^ mice, Smad3 was activated in both perivascular area and neointima, which was overlapped in Pn-positive cells (Fig 4A). On the other hand, in Pn-Cre/*Tgfbr1*^fl/fl^ mice, Smad2/3 was inhibited in neointima but Smad2/3 activity was not inhibited in Pn-negative cells (Fig 4B). Immunostaining for TGF-β receptor type I (Tgfbr1) showed Tgfbr1 protein reduction in perivascular area of pulmonary artery in Pn-Cre/*Tgfbr1*^fl/fl^ mice (S2E and S2F Fig). These results indicate that Pn-Cre mediated knockdown of TGF-β signaling is achieved in this model.

### Inhibition of pulmonary hypertension in Pn-Cre/*Tgfbr1*^fl/fl^ mice models

RV pressure was significantly decreased in Pn-Cre/*Tgfbr1*^fl/fl^ mice compare to *Tgfbr1*^fl/fl^ mice under hypoxic condition (Fig 4C). There was no significant difference in either RV dP/dt max and RVW/LVW between the two groups (Fig 4D and 4E). Medial thickening of PA shown as PA muscularization was significantly attenuated in Pn-Cre/*Tgfbr1*^fl/fl^ mice (Fig 4F).

## Discussion

In the present study, we demonstrated that Pn was expressed in remodeled PA specifically under hypoxic conditions in a mice model, and its expression was distributed in neointima and perivascular areas of remodeled PA. Furthermore, in Pn-Cre/*Tgfbr1*^fl/fl^ mice, Pn expression was suppressed and Smad3 activation attenuated in remodeled PA, resulting in inhibition of RV pressure elevation and medial thickening of PA. These findings suggest that TGF-β signaling in Pn expressing cell may have an important role in pathogenesis of PAH by controlling medial thickening.

Progressive PA wall thickening and the obliteration caused by neointimal formation are attributable to increased proliferation and migration of cells considered to be SMCs, because they are α-SMA positive cells, but the origin of these cell is unclear[24,25]. There are various reports of their origin, these cells have been considered to originate as stem cells[26], fibrocytes[27], or transform from endothelial cells[28,29]. Our study showed that Pn is strongly involved in the formation of remodeled PA, indicating that not only PASMCs, but also various cells including ECM proteins such as Pn, are important for the pathogenesis of PAH. Indeed, Pn is thought to work as a multifunctional modulator of cell processes underlying the proliferative and remodeling phases of healing[8]. In addition, it has been reported that treatment of mouse fibrocytes or fibroblasts with Pn leads to increased TGF-β production and vice versa, indicating that Pn and TGF-β are co-regulate each other[30]

Since Pn was first identified as cell adhesion protein in 1993, many studies have been conducted on it[31]. Pn is understood to have many functions in osteogenesis, tissue repair, cancer growth, the cardiovascular and respiratory systems, in various inflammatory settings[32]. However, there have been few reports on the role of Pn in PAH, and its involvement in PAH pathophysiology is poorly understood. Li et al. found that Pn mRNA levels are increased in rat lung and that hypoxic stress induces Pn expression in isolated rat PASMCs *in vitro*[9,33]. In addition, Abdul-Salam et al. demonstrated using proteomic analysis that Pn levels in lung tissues from patients with PAH was markedly increased and localized in neointima of remodeled PA[10]. Consistent with these findings, we found that Pn expression was increased by hypoxic stress in our mouse model, and that Pn was mainly expressed in neointima and perivascular area, suggesting that Pn is deeply involved in the pathology of PAH due to hypoxia. Concerning which cells in the lung express Pn in PAH, Abdul-Salam et al. reported that Pn is localized in the submucosa of bronchi and bronchioles and distributed in the alveolar walls histologically in patients with PAH. Additionally, Pn immunostaining is prominent in the airways and in remodeled PA, typically found in the neointimal rather than medial layer, and mainly displays a distribution pattern different to that of α-SMA[10]. Our co-immunostaining studies showed Pn-expressed cells were mixed cell types of fibroblasts, pericytes and PASMCs. It has been reported that Pn is secreted not only from fibrocytes or fibroblasts but also from PASMCs in the lung under stressed condition[9,30,34,35]. Given that Pn is a marker for proliferation in these cells[36,37,38], Pn-positive cells would be responded cells for the pathological stress and have a pivotal role on perivascular cellular proliferation and vascular remodeling in the lung with PAH. Pn-expressing cell-targeted inhibition of TGF-β signaling (Pn-Cre/*Tgfbr1*^fl/fl^ mice) effectively attenuated the development of PAH under hypoxic condition, suggesting that Pn-positive cells are crucial for TGF-β-mediated vascular remodeling in PAH.

TGF-β superfamily growth signals may contribute to the pathogenesis of PAH in both PASMCs[39,40] and fibroblasts[41]. Recently, studies of fibrotic disease have shown that fibroblasts form patients with idiopathic pulmonary fibrosis are producing Pn and TGF-β induces Pn secretion from lung mesenchymal cells[35]. TGF-β1 and Pn have also been shown to co-regulate each other in fibroblasts of a mouse model[30]. These reports prove that Pn and TGF-β are closely related to the proliferation and degeneration of lung fibroblasts. In the present study, Smad2/3 activation in Pn expressing cells was attenuated in Pn-Cre/*Tgfbr1*^fl/fl^ mice under hypoxic condition. RV pressure in these mice was significantly decreased, and PA muscularization was attenuated compared with control mice. These results suggest that Smad2/3-mediated TGF-β signaling in Pn-expressing cells plays an important role in the pathogenesis of PAH by controlling medial thickening.

## Conclusions

In conclusion, under hypoxic conditions, the activation of TGF-β signaling in Pn-expressing cells was down-regulated and hypoxia-induced PAH was attenuated in Pn-Cre/*Tgfbr1*^fl/fl^ mice. Given that Pn expression is promoted in a remodeled PA-specific manner, targeting TGF-β signaling in Pn-expressing cells is a promising therapeutic strategy for PAH.

## Supporting information

S1 Fig Hypoxia-induced murine PAH model. RV catheterization under normoxic and 4-weeks hypoxic condition (**A**-**C**). Representative RV pressure measurements are displayed in **A**. Bar charts show RVESP, RVdP/dtmax, and indices of RV weight (RVW/LVW) in normoxia control (n = 8) and 4 weeks hypoxia (n = 5) (**B**-**D**). Pulmonary arteries were considered muscularized if they had a distinct double-elastic lamina visible throughout the diameter of the vessel cross section with Elastica Van Gieson staining (**E**). The percentage of vessels with double-elastic lamina was calculated as the number of muscularized vessels per total number of vessels in normoxia control (n = 8) and 4 weeks hypoxia (n = 5) groups, showing PA muscularization was significantly increased in the hypoxia group (**F**). Group comparisons were performed by unpaired 2-tailed Student’s t test. RV, right ventricle; ESP, end-systolic pressure; EDP, end-diastolic pressure; RVW, right ventricle weight; LVW, left ventricle weight; PA, pulmonary artery.
(TIF)

S2 Fig TGF-β type I receptor immunostaining. Pn-Cre expression induced by hypoxia (**A**-**D**). Under normal oxygen condition (normoxia), lung tissues of Pn-Cre/lacZ reporter mice showed low LacZ expression (**A** and **B**). Size bar: 50μm (**A**), 20μm (**B**); Under chronic hypoxia, lung tissues of Pn-Cre/lacZ reporter mice showed higher LacZ expression (**C** and **D**). LacZ and αSMA were partially overlapped. Panel **C** shows LacZ-positive and αSMA -positive vessel. Panel **D** shows LacZ-positive but αSMA -negative vessel. Size bar: 20μm (**C** and **D**); Immunostaining shows TGF-β type I receptor (Tgfbr1) expression in lung tissues of *Tgfbr1*^fl/fl^ mice (**E**) and Pn-Cre/*Tgfbr1*^fl/fl^ mice (**F**). Tgfbr1 was reduced in perivascular area in Pn-Cre/*Tgfbr1*^fl/fl^ mice. *, pulmonary artery; †, bronchus.
(TIF)
