# Supplementary figures and images for: Periostin-expressing cell-specific transforming growth factor-β inhibition in pulmonary artery prevents pulmonary arterial hypertension
Source: PLoS One. 2019 Aug 22;14(8):e0220795. doi: 10.1371/journal.pone.0220795 (PMC6705784; doi:10.1371/journal.pone.0220795)

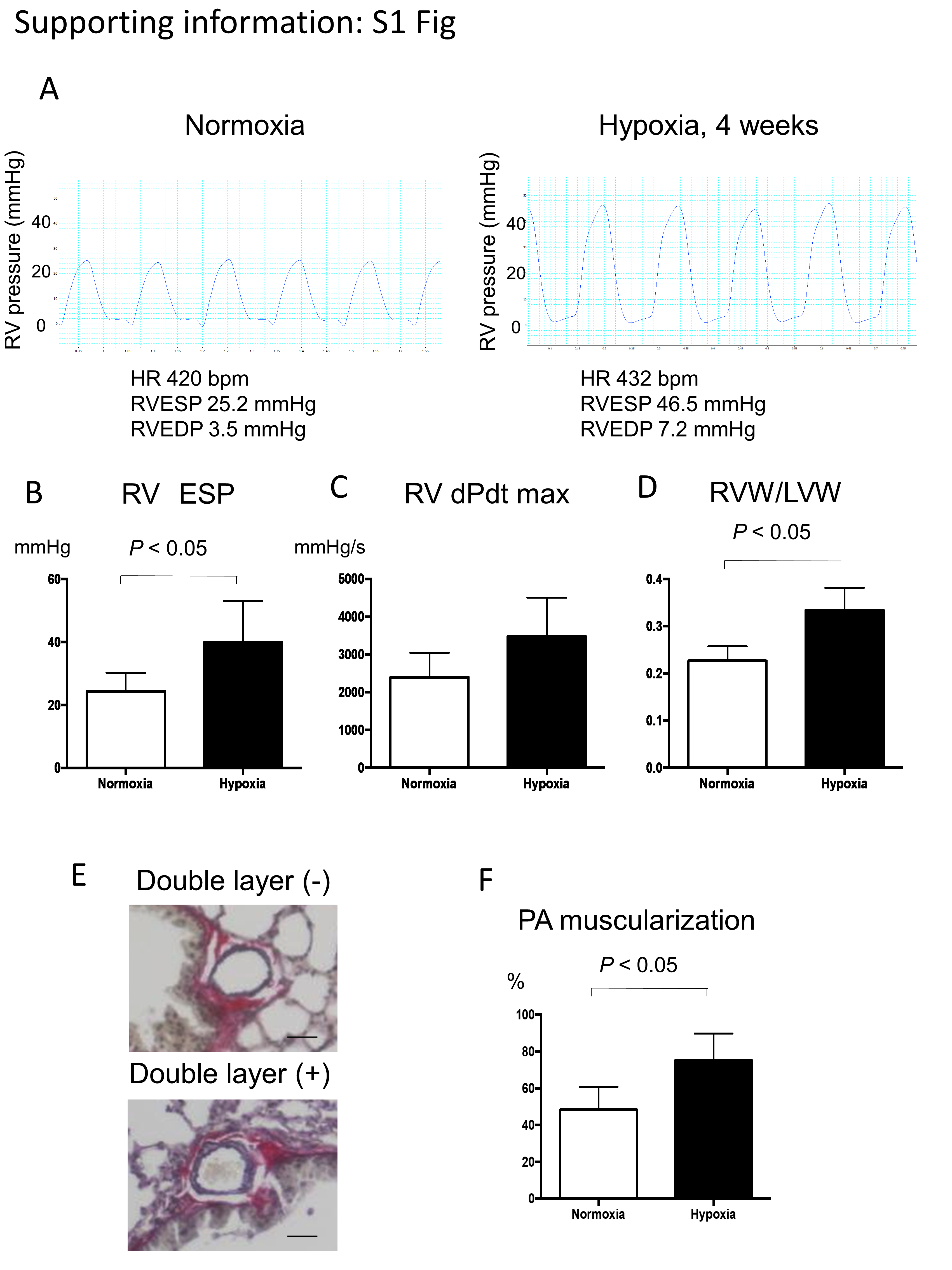

Supplement: S1 Fig — RV catheterization under normoxic and 4-weeks hypoxic condition (A-C). Representative RV pressure measurements are displayed in A. Bar charts show RVESP, RVdP/dtmax, and indices of RV weight (RVW/LVW) in normoxia control (n = 8) and 4 weeks hypoxia (n = 5) (B-D). Pulmonary arteries were considered muscularized if they had a distinct double-elastic lamina visible throughout the diameter of the vessel cross section with Elastica Van Gieson staining (E). The percentage of vessels with double-elastic lamina was calculated as the number of muscularized vessels per total number of vessels in normoxia control (n = 8) and 4 weeks hypoxia (n = 5) groups, showing PA muscularization was significantly increased in the hypoxia group (F). Group comparisons were performed by unpaired 2-tailed Student’s t test. RV, right ventricle; ESP, end-systolic pressure; EDP, end-diastolic pressure; RVW, right ventricle weight; LVW, left ventricle weight; PA, pulmonary artery. (TIF) [file pone.0220795.s001.TIF]

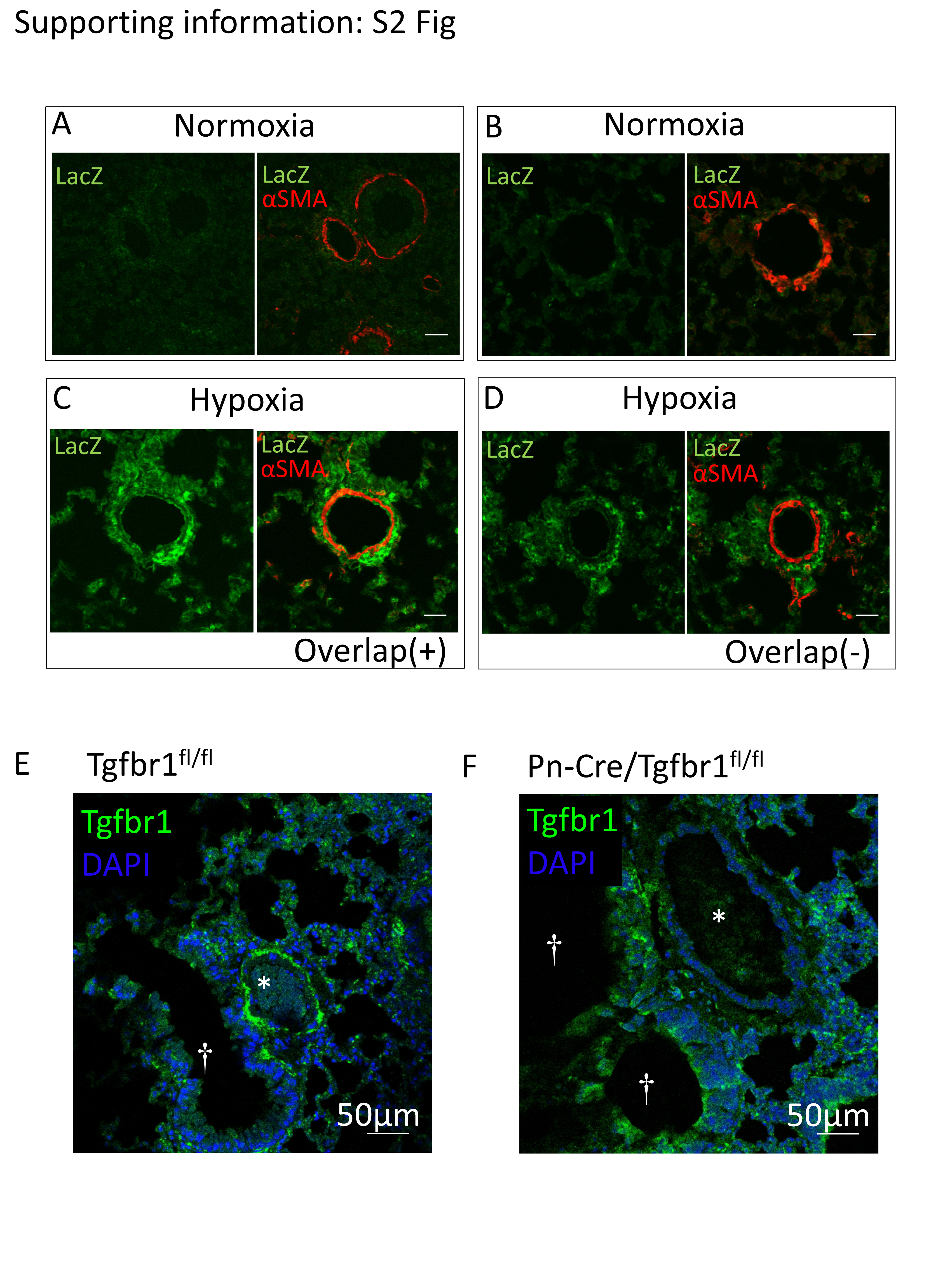

Supplement: S2 Fig — Pn-Cre expression induced by hypoxia (A-D). Under normal oxygen condition (normoxia), lung tissues of Pn-Cre/lacZ reporter mice showed low LacZ expression (A and B). Size bar: 50μm (A), 20μm (B); Under chronic hypoxia, lung tissues of Pn-Cre/lacZ reporter mice showed higher LacZ expression (C and D). LacZ and αSMA were partially overlapped. Panel C shows LacZ-positive and αSMA -positive vessel. Panel D shows LacZ-positive but αSMA -negative vessel. Size bar: 20μm (C and D); Immunostaining shows TGF-β type I receptor (Tgfbr1) expression in lung tissues of Tgfbr1fl/fl mice (E) and Pn-Cre/Tgfbr1fl/fl mice (F). Tgfbr1 was reduced in perivascular area in Pn-Cre/Tgfbr1fl/fl mice. *, pulmonary artery; †, bronchus. (TIF) [file pone.0220795.s002.TIF]
